# Towards Electrochemical Sensor Based on Molecularly Imprinted Polypyrrole for the Detection of Bacteria—*Listeria monocytogenes*

**DOI:** 10.3390/polym15071597

**Published:** 2023-03-23

**Authors:** Viktorija Liustrovaite, Maksym Pogorielov, Raimonda Boguzaite, Vilma Ratautaite, Almira Ramanaviciene, Greta Pilvenyte, Viktoriia Holubnycha, Viktoriia Korniienko, Kateryna Diedkova, Roman Viter, Arunas Ramanavicius

**Affiliations:** 1 Department of Physical Chemistry, Faculty of Chemistry and Geosciences, Vilnius University, Naugarduko Str. 24, LT-03225 Vilnius, Lithuania; 2 Biomedical Research Centre, Sumy State University, R-Korsakova Street, 40007 Sumy, Ukraine; 3 Institute of Atomic Physics and Spectroscopy, University of Latvia, Jelgavas iela 3, LV-1004 Riga, Latvia; 4 Department of Nanotechnology, State Research Institute Center for Physical Sciences and Technology, Saulėtekio Av. 3, LT-10257 Vilnius, Lithuania; 5 NanoTechnas-Center of Nanotechnology and Materials Science, Institute of Chemistry, Faculty of Chemistry and Geosciences, Vilnius University, Naugarduko Str. 24, LT-03225 Vilnius, Lithuania

**Keywords:** molecularly imprinted polymer, molecularly imprinted polypyrrole, *Listeria monocytogenes*, whole-cell imprinting, pulsed amperometric detection, template extraction method, trypsin, L-lysine, acetic acid, sulphuric acid

## Abstract

Detecting bacteria—*Listeria monocytogenes*—is an essential healthcare and food industry issue. The objective of the current study was to apply platinum (Pt) and screen-printed carbon (SPCE) electrodes modified by molecularly imprinted polymer (MIP) in the design of an electrochemical sensor for the detection of *Listeria monocytogenes*. A sequence of potential pulses was used to perform the electrochemical deposition of the non-imprinted polypyrrole (NIP-Ppy) layer and *Listeria monocytogenes*-imprinted polypyrrole (MIP-Ppy) layer over SPCE and Pt electrodes. The bacteria were removed by incubating Ppy-modified electrodes in different extraction solutions (sulphuric acid, acetic acid, L-lysine, and trypsin) to determine the most efficient solution for extraction and to obtain a more sensitive and repeatable design of the sensor. The performance of MIP-Ppy- and NIP-Ppy-modified electrodes was evaluated by pulsed amperometric detection (PAD). According to the results of this research, it can be assumed that the most effective MIP-Ppy/SPCE sensor can be designed by removing bacteria with the proteolytic enzyme trypsin. The LOD and LOQ of the MIP-Ppy/SPCE were 70 CFU/mL and 210 CFU/mL, respectively, with a linear range from 300 to 6700 CFU/mL.

## 1. Introduction

*Listeria monocytogenes* infections with Gram-positive, rod-shaped bacteria with an optimum growing temperature at 37 °C [1] are among the leading causes of foodborne illness-related mortality [2]. *Listeria monocytogenes* is an environmental contaminant that primarily inhabits soil. Various animals (ruminants, birds, marine life, insects, ticks, and crustaceans) are carriers of bacteria [3]. *Listeria monocytogenes* can enter the food supply chain and contaminate a wide variety of food products, including meat products; raw, unpasteurised milk and cheeses; ice cream; raw or processed vegetables; raw or processed fruits; raw or undercooked poultry, sausages, hot dogs, and deli meats; and raw or smoked fish and other seafood [4].

One type of sickness induced by *Listeria monocytogenes* is very dangerous and can result in septicaemia and meningitis, with a case-fatality rate of 20–30% [5]. Another kind of disease caused by *Listeria monocytogenes* is a non-invasive gastrointestinal ailment that typically has no consequences. However, despite the low-level incidences of listeriosis in the general population, it remains a significant and deadly food-borne disease with a hospitalisation rate of over 95% [6]. The major problem is that *Listeria monocytogenes* affects vulnerable segments of populations, including the elderly, pregnant women, unborn babies, and immunocompromised people (patients with cancer or AIDS, or after organ transplantations) [7]. Thus, pregnant women have a 17-fold increased risk of contracting invasive listeriosis [8], and the mortality associated with *Listeria monocytogenes* infection is responsible for 22% of fatalities in immunocompromised adults [4].

Detecting *Listeria monocytogenes* is an essential healthcare and food industry issue [9]. The minimal infection dose for listeriosis is 100 colony-forming units per gram (CFU/g) of food. The majority of countries have zero tolerance towards the presence of *Listeria monocytogenes* in food [10]. The European Regulation on Microbiological Criteria for Foodstuffs does not allow the presence of *Listeria monocytogenes* in foods for infants and particular medical purposes. However, all food can have 100 CFU/g of the organism during its shelf life [11]. In this case, fast and precise detection of *Listeria monocytogenes* is required both for the healthcare and food industries.

Among many methods for the identification of *Listeria monocytogenes,* colony plate counting is accepted to be the ‘gold standard’ procedure [12]. The detection of *Listeria monocytogenes* has been proposed using several standard techniques, including surface plasmon resonance [13], quartz crystal microbalance [14], and enzyme-linked immunosorbent assay (ELISA) [15,16]. These methods are crucial and can essentially meet the criteria for *Listeria monocytogenes* detection. However, they often have shortcomings and are labour-intensive, time-consuming, or complicated. In ELISA, secondary antibodies connected to an enzyme are immobilised in a well to capture *Listeria* antigens. These tests are used in food testing because they are straightforward, simple to interpret, and do not require much sample handling. However, they produce results in roughly 30–50 h and are not as sensitive as molecular methods. This technique has a sensitivity range of approximately 10^5^–10^6^ CFU/mL [17]. The electrochemical approach, in comparison, is straightforward, sensitive, time-saving, inexpensive, and simple to use, giving it several distinct benefits over the other methods. Many excellent electrochemical systems have been successfully built in recent years to detect *Listeria monocytogenes* [18,19,20,21], including antibody- or DNA-based methods [1,12].

The exceptional selectivity of molecularly imprinted polymers towards molecularly imprinted analytes makes them appealing. Molecular imprinting can create a binding site uniquely suited to a specific molecule [22]. The molecular imprinting approach enables the development of particular molecular recognition sites that work on the idea of complementarity between the imprinted sites and the analyte due to its many distinctive benefits, including simplicity in production, affordability, and excellent stability [23,24]. Therefore, MIPs can specifically bind the analytes of interest that serve as templates for their development [25,26,27,28,29]. However, due to the size, imprinting the whole cell in the polymers is exceptionally challenging [30,31]. Several studies have evaluated the suitability of MIP-based sensors for detecting *Listeria monocytogenes* bacteria [32,33,34]. Mainly two factors governing the recognition of *Listeria monocytogenes* bacteria should be taken into account: (i) discrimination of the bacteria by their cell shape (e.g., round or rod-shaped bacteria, namely *Staphylococcus aureus* or *Escherichia coli*) and (ii) chemical recognition due to the interaction of functional groups present in polymers with functional groups that are localised on the surface of the cell, e.g., cis-diol groups of the glycan chains [35]. Taking into account the emerging problems, Piletsky et al. [30] raised some questions related to the materials suitable for the modification of electrodes by MIP-based layers. The authors of that study concluded that “success in this area will result in new paradigms for MIP applications that both complement existing therapeutic and disposal or reuse in field diagnostic techniques”.

The current research sought to develop a MIP-based sensor for detecting *Listeria monocytogenes.* Firstly, the goal was to test the performance of two electrodes. Pt and SPCE were modified with a polypyrrole layer made from the polymerisation solution of *Listeria monocytogenes* bacteria and pyrrole dissolved in phosphate-buffered saline (PBS), pH 7.4. A novelty of this study or a second approach was to determine the most efficient solution for extraction. Thus, *Listeria monocytogenes* bacteria from imprinted cavities were extracted using trypsin and L-lysine and compared with more conventional extraction methods such as sulphuric and acetic acids. Herein, the analytical performance of electrodes modified by a non-imprinted polypyrrole (NIP-Ppy) layer and the electrodes modified with the MIP-Ppy layer were compared. Thirdly, the sensitivity (LOD and LOQ) and repeatability criteria were effectively employed to detect *Listeria monocytogenes*.

## 2. Materials and Methods

### 2.1. Materials and Electrochemical Measurements

*Listeria monocytogenes* were obtained from the Bacteria Collection of Sumy State University (Sumy, Ukraine). To preserve the antigenic structure on the *Listeria monocytogenes* membrane but eliminate virulence, 10^9^ CFU/mL bacteria were immersed in 70% ethanol and placed under UV light for 24 h. This procedure allows the destruction of bacterial DNA with minimal influence on the cell wall and shape, which are necessary for MIP development.

Pyrrole 98% (CAS# 109-97-7, Alfa Aesar, Kandel, Germany), phosphate-buffered saline (PBS) tablets, pH 7.4 (CAS# 7647-14-5, Sigma-Aldrich, Steinheim, Germany), sulphuric acid (96%, CAS# 7664-93-9, Lachner, Neratovice, Czech Republic), acetic acid (99.8%, CAS# 64-19-7, 99.8%, Lachner, Czech Republic), trypsin 500 U/mL (TrypZean^®^ Solution, Sigma-Aldrich, SKU T3449-500 ML), and 0.1% (*w*/*v*) L-lysine solution in H_2_O (Sigma-Aldrich, CAS# 25988-63-0) were used as received for bacteria removal from the Ppy-matrix to form the MIP-Ppy layer. All reagents were of analytical grade and were used without additional purification. All aqueous solutions were prepared in deionised water.

Electrochemical characterisation of the working surfaces was performed using two systems. A potentiostat/galvanostat AUTOLAB TYPE III (ECO-Chemie, Utrecht, The Netherlands) operated by FRA2-EIS ECO-Chemie software (ECO-Chemie, Utrecht, The Netherlands) was used for the first electrochemical system. The first set of electrodes, DRP-110 screen-printed carbon electrodes (SPCEs), which are based on a working electrode with a geometric area of 0.126 cm^2^, a carbon-based counter electrode, and an Ag/AgCl-based reference electrode, was purchased from Metrohm DropSens (Oviedo, Spain).

For the second electrochemical system, the second set of electrodes was based on (i) a Pt disk with a 1 mm diameter sealed in glass as the working electrode, (ii) an Ag/AgCl in 3 M KCl solution electrode as a reference electrode (Ag/AgCl), and (iii) a Pt disk of 2 mm diameter as a counter electrode. Measurements were done in a home-made cell with a total volume of 300 µL, and electrochemical characterisation was performed using a portable potentiostat controlled by DStat interface software from Wheeler Microfluidics Lab (University of Toronto, Toronto, ON, Canada).

Scanning electron microscope (SEM) images were obtained with a scanning electron microscope (Hitachi-70 S3400 N VP-SEM).

### 2.2. Pre-Treatment of Working Electrodes

Pre-treatment of electrodes for the first electrochemical system: Before the electrochemical deposition of Ppy, the working electrodes underwent pre-treatment. A potential cycling approach was used to electrochemically clean SPCEs. The cleaning was carried out in 0.5 M sulphuric acid by 20 potential cycles in a potential range between −100 mV and +1200 mV vs. Ag/AgCl at a potential sweep rate of 100 mV/s.

Pre-treatment of electrodes for the second electrochemical system: The Pt electrode was pre-treated before electrochemical deposition following the procedure described in previous studies [36]. All solutions were thoroughly degassed just before use with a stream of nitrogen (N_2_). According to this procedure, the Pt electrode was rinsed with concentrated HNO_3_ solution in an ultrasonic bath for 10 min, then rinsed with water and polished with alumina paste. Later, it was rinsed with water again and then with a solution of 10 M NaOH, then with a 5 M sulphuric acid solution in an ultrasonic bath for 5 min. Electrochemical electrode cleaning was carried out in 0.5 M sulphuric acid by cycling the potential 20 times in the range between −100 mV and +1200 mV vs. Ag/AgCl at a sweep rate of 100 mV/s. The assessment of the bare electrode surface was performed by cyclic voltammogram. To improve the adhesion of the Ppy layer to the electrode surface, a layer of ‘platinum black’ was formed over the working electrode. The deposition of ‘platinum black’ clusters was performed in a solution of 5 mM H_2_PtCl_6_ containing 0.1 M of KCl by 10 potential cycles in the range between +500 mV and −400 mV vs. Ag/AgCl at a potential sweep rate of 10 mV/s.

### 2.3. Electrochemical Modification of Electrodes by NIP-Ppy and MIP-Ppy Layers

The polymerisation solution contained 0.5 M of pyrrole in PBS and was used to electrochemically deposit the NIP-Ppy layer [25]. The deposition of MIP-Ppy on Pt and SPCE electrodes was performed in several steps: (i) during the first step, the Ppy under-layer was electrochemically deposited from polymerisation solution containing a 0.5 M solution of pyrrole, and a sequence of 5 potential pulses (of +950 mV for 1 s and 0 V for 30 s) was applied [29,36]; (ii) during the deposition of the second layer, 10^9^ CFU/mL of *Listeria monocytogenes* bacteria was additionally added into the same polymerisation bulk solution and again the sequence of 5 potential pulses (of +950 mV for 1 s and 0 V for 30 s) was applied; (iii) the purpose of the third step was to remove imprinted bacteria from the formed Ppy layer by incubating electrodes in different extraction solutions to form the MIP-Ppy (0.05 M sulphuric acid, 10% acetic acid, 0.1% L-lysine, 10 U/mL trypsin for at 37 °C for 30 min). The NIP-Ppy-based layer was formed similarly to MIP-Ppy (only bacteria were not added), and the abovementioned extraction solutions similarly treated the NIP-Ppy-modified electrode.

Pulsed amperometric detection was used to assess MIP-Ppy- and NIP-Ppy-modified electrodes utilising a sequence of 10 potential pulses of +600 mV vs. Ag/AgCl lasting for 2 s, and 0 V vs. Ag/AgCl for 2 s.

**Scheme 1:**
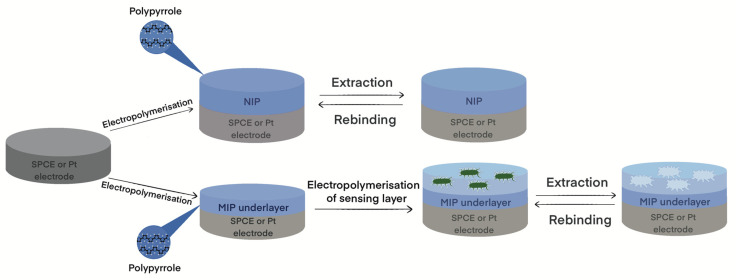
Schematic representation of electrode modification.

The limit of detection (LOD) and limit of quantification (LOQ) were calculated according to Equations (1) and (2):

(1) LOD =3.3 σ/S

(2) LOQ =10 σ/S

where σ is the standard deviation and S is the slope of the linear relationship on the calibration plot. A schematic representation of electrode modification is presented in Scheme 1.

## 3. Results

### 3.1. Electrodeposition of Molecularly Imprinted Polypyrrole

**Figure 1:**
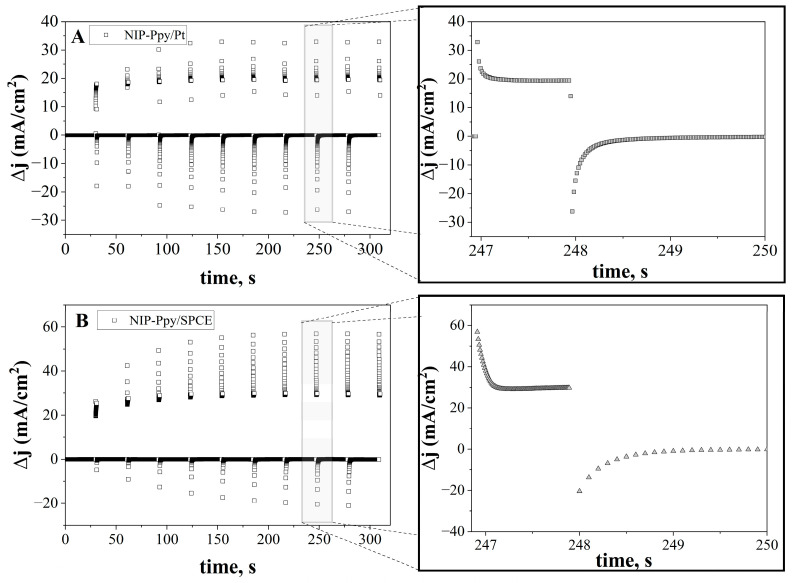
Electrochemical deposition of NIP-Ppy layers: (**A**) on Pt electrodes (second electrochemical system); (**B**) on SPCE electrodes (first electrochemical system), showing the profile of the current registered during the formation of the NIP-Ppy layer from polymerisation solution without any bacteria. Insets—extended profile of the current registered during the indicated potential pulse.

**Figure 2:**
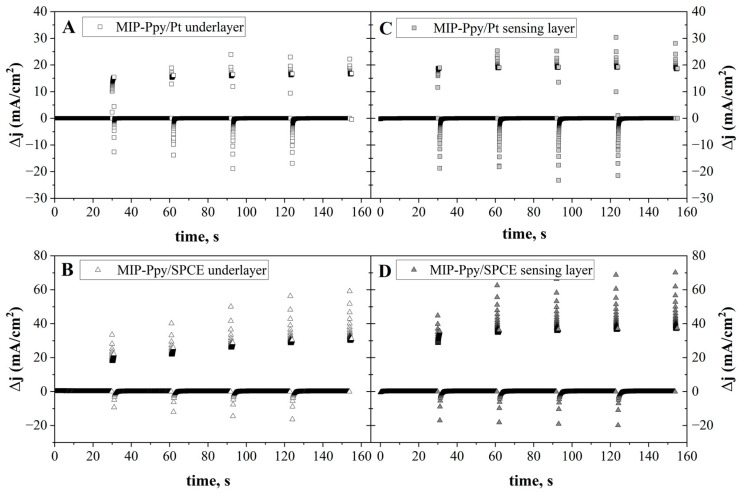
Current profiles registered during the deposition of Ppy underlayers (**A**) on Pt electrodes (second electrochemical system), and (**B**) on SPCE electrodes (first electrochemical system). Current profiles registered during electrochemical deposition of the polypyrrole layers with entrapped *Listeria monocytogenes* bacteria (**C**) on Pt electrodes (2nd electrochemical system), and (**D**) on SPCE electrodes (1st electrochemical system).

MIP-Ppy and NIP-Ppy layers were electrochemically deposited on the surface of Pt and SPCE electrodes using a series of potential pulses. Figure 1A,B depict the profile of the potential pulse series during the deposition of the NIP-Ppy layer on Pt and SPCE electrodes, respectively. The electrochemical formation of the MIP-Ppy layer on Pt and SPCE electrodes was performed in several steps as described in the experimental section, respectively (Figure 2A,B). The first step was based on the electrodeposition of the Ppy-based under-layer to support and cover the electrode. This Ppy-based under-layer decreased the direct interaction of *Listeria monocytogenes* with the electrode surface before forming the MIP-Ppy sensing layer. The deposited thin Ppy under-layer effectively favoured the formation of the MIP-Ppy-sensing layer during the second sensing-layer-formation step. The entrapped *Listeria monocytogenes* bacteria acted as a template in an upper Ppy layer (sensing layer), which, after the removal of bacteria, formed the MIP-Ppy layer. Electrochemical Ppy deposition enabled control of the thickness of formed layers and entrap the *Listeria monocytogenes* bacteria templates in the electropolymerised matrix. The entrapped *Listeria monocytogenes* bacteria templates were removed from the MIP-Ppy layer by incubation in several extraction solutions.

### 3.2. Extraction of Imprinted Bacteria from the MIP-Ppy Layer

**Figure 3:**
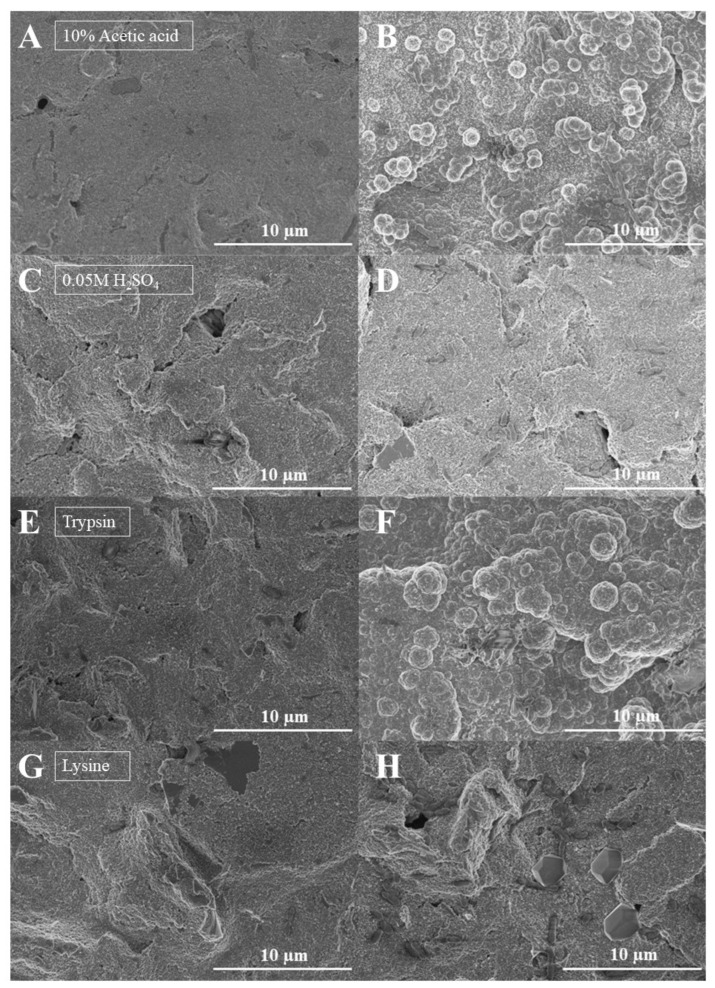
SEM images of NIP-Ppy/SPCE (**A**,**C**,**E**,**G**) and MIP-Ppy/SPCE (**B**,**D**,**F**,**H**) electrodes after incubation in different template extraction solutions: (**A**,**B**) 10% acetic acid, (**C**,**D**) 0.05 M sulphuric acid, (**E**,**F**) 10 U/mL trypsin, (**G**,**H**) 0.1% L-lysine at 37 °C for 30 min.

Several different extraction solutions were used to remove *Listeria monocytogenes* bacteria from the MIP-Ppy layer formed on the SPCE. The first template extraction method was based on the incubation of NIP-Ppy/SPCE, and MIP-Ppy/SPCE acetic acid solution was applied. Acetic acid is a weak organic acid harmful to most bacteria, even at concentrations as low as 0.5 wt%. Acetic acid, among other harmful effects, leads to a drop in intracellular pH and the disruption of some metabolic chains by acetic acid anion [37]. Figure 3B represents the bacteria cells after incubation in acetic acid. As a result, the cell membrane develops holes that allow the cytosol and cytoplasmic organelles to leak out. The rough, uneven pits on the bacteria cell surface showed that the extraction by acetic acid was highly effective. However, the incubation of NIP-Ppy/SPCE and MIP-Ppy/SPCE in a sulphuric acid-containing solution revealed that the surface of formed NIP-Ppy and MIP-Ppy (Figure 3C,D) seem identical.

The third template extraction solution used to remove the *Listeria monocytogenes* bacteria template from MIP-Ppy/SPCE was an enzyme (trypsin) solution. Trypsin catalyses the hydrolysis of cell wall proteins to form peptides. In addition, we tried to remove *Listeria monocytogenes* bacteria from MIP-Ppy/SPCE by L-lysine, which is a zwitterion amino acid and was expected to be efficient for the dissociation and removal of bacteria from the Ppy-based matrix. However, registered results (Figure 3H) illustrate that the L-lysine-based bacteria extraction procedure was not efficient compared to that based on trypsin. Moreover, L-lysine is crucial for protein synthesis and is also present in the peptidoglycan layer on the cell walls of Gram-positive bacteria; therefore, it supports cell metabolism. Additionally, it should be noted that trypsin has a specific target in the cell wall and does not affect the Ppy layer; this effect would be an extra advantage for the removal of bacteria-based templates and the development of MIP-Ppy-based sensor platforms. Trypsin is a well-known pancreatic enzyme that digests proteins by specifically hydrolysed peptide bonds C-terminal to the amino acid residues of lysine (Lys) and arginine (Arg) [38]. Some studies have shown increased levels of proteolytic enzymes, including trypsin in inflammatory sites, followed by bacterial lysis. For example, Grenier demonstrated that Gram-positive bacteria from the oral cavity are more resistant to lysis than Gram-negative bacteria [39]. Meanwhile, Zhou et al. showed the same effect of the enzyme on both bacteria types (including biofilm formation) in a concentration of 2 mg/mL [40].

In contrast to trypsin, L-lysine is an amino acid that, at pH = 7.0, is a zwitterion; therefore, we expected that it can act as an efficient agent for the dissociation/removal of some compounds from polymeric structures. As demonstrated by this research, 10 U/mL trypsin solution is the most efficient for *Listeria monocytogenes* cell removal from the Ppy layer and to form MIP-Ppy.

### 3.3. Electrochemical Characterisation of Bacteria-Imprinted MIP-Ppy Layer

**Figure 4:**
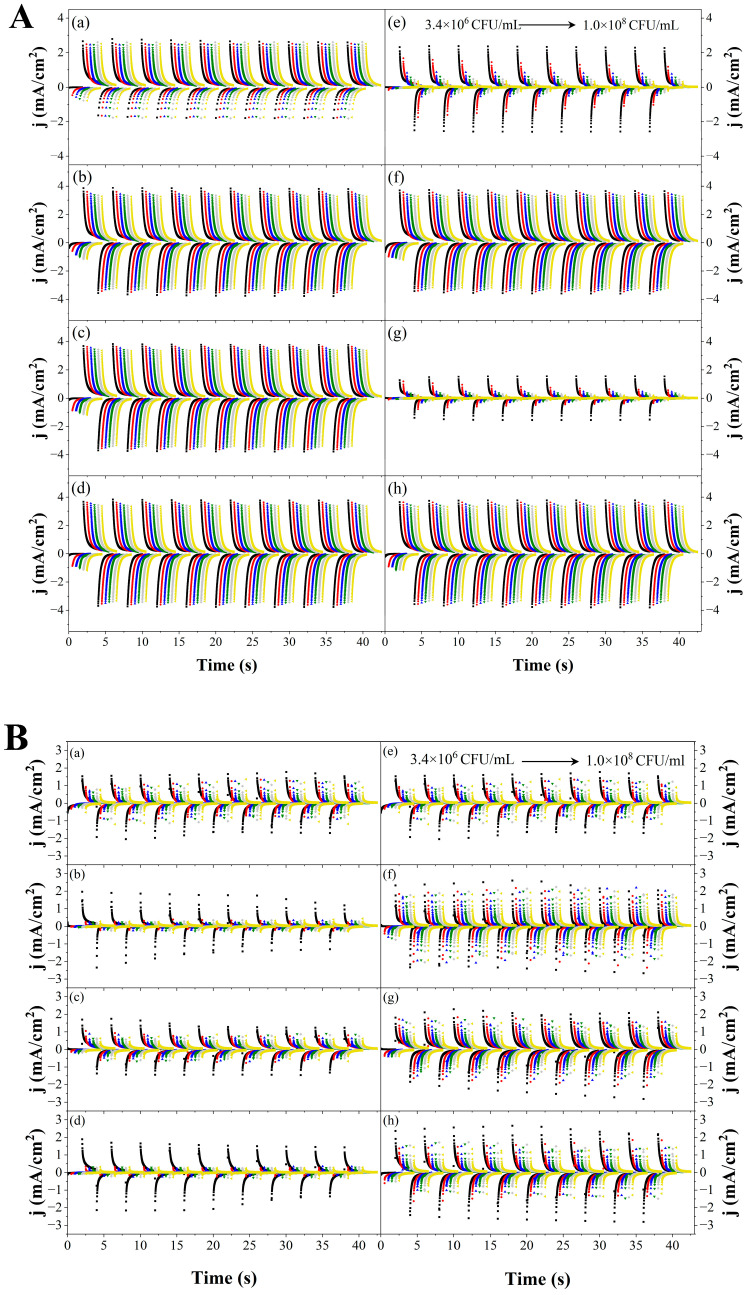
Current density vs. time determined by NIP-Ppy/SPCE (**a**–**d**), MIP-Ppy/SPCE (**e**–**h**) electrodes (**A**) and NIP-Ppy/Pt (**a**–**d**), MIP-Ppy/Pt (**e**–**h**) (**B**), with a concentration range of 3.4 × 10^6^, 1.0 × 10^7^, 2.3 × 10^7^, 4.0 × 10^7^, 6.7 × 10^7^, 1.0 × 10^8^ CFU/mL *Listeria monocytogenes* bacteria, prepared using different extraction solutions:10% acetic acid (**a**,**e**), 0.05 M sulphuric acid (**b**,**f**), 10 U/mL trypsin (**c**,**g**), and 1% L-lysine (**d**,**h**) to remove *Listeria monocytogenes* bacteria from imprinted cavities.

The formed NIP-Ppy and MIP-Ppy layers were assessed using pulsed amperometric detection to assess the current density in a sequence of 10 potential pulses of +600 mV for 2 s and 0 mV for 2 s. The determination of *Listeria monocytogenes* bacteria at several different concentrations was performed using two different electrochemical systems (Figure 4). MIP-Ppy- and NIP-Ppy-modified SPCE electrodes (Figure 4A) and MIP-Ppy- and NIP-Ppy-modified Pt electrodes (Figure 4B) were incubated in a PBS solution, pH 7.4, in a concentration range of 3.4 × 10^6^–1.0 × 10^8^ CFU/mL *Listeria monocytogenes* bacteria. Figure 4 shows the dependence of the amperometric response. During the analysis, a decrease in the current with increasing bacteria concentration was observed, as usual in the redox-inactive analytes [41].

#### 3.3.1. Assessment of first Electrochemical System

**Figure 5:**
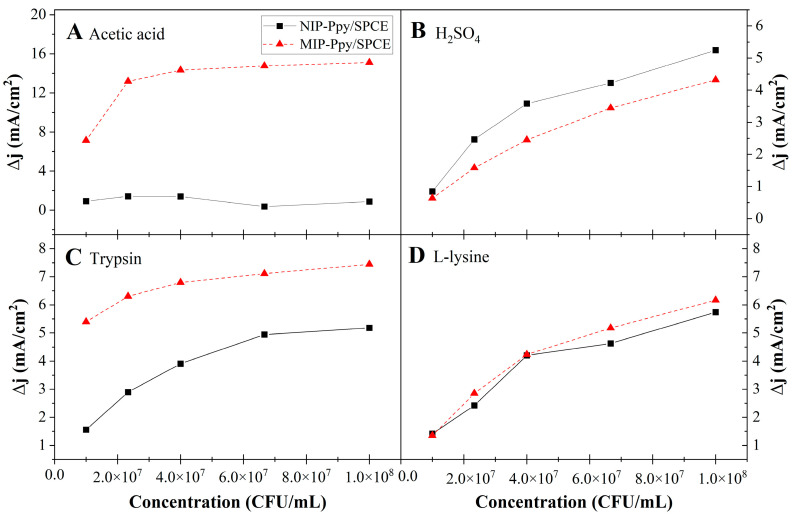
The current density of NIP-Ppy/SPCE (solid black lines) and MIP-Ppy/SPCE (dashed red lines) electrodes registered using pulsed amperometric detection after incubation in solutions containing different *Listeria monocytogenes* bacteria concentrations; *Listeria monocytogenes* from MIP-Ppy was extracted using different extraction solutions: (**A**) 10% acetic acid, (**B**) 0.05 M of sulphuric acid, (**C**) 10 U/mL of trypsin, (**D**) 0.1% L-lysine.

Assessment of MIP-based sensor towards imprinted *Listeria monocytogenes* bacteria was performed in the concentration range from 0 to 10^8^ CFU/mL. First, *Listeria monocytogenes* bacteria were eliminated from imprinted cavities using several extraction solutions, namely 10% acetic acid (Figure 5A), 0.05 M of sulphuric acid (Figure 5B), 10 U/mL of trypsin (Figure 5C), and 0.1% L-lysine (Figure 5D). As we can see from Figure 4, acetic acid was highly effective, as the current density of MIP-Ppy/SPCE was at least 12 times higher than that of NIP-Ppy/SPCE. While using the sulphuric acid solution, we observed only a slight change in the current density. However, the acid tolerance of *Listeria monocytogenes* bacteria is a predicted molecular response, which ensures cell survival in an unfavourable environment. The increased intracellular survival and the development of acid-adapted *Listeria monocytogenes* cells in the vacuoles and cytoplasm were confirmed by morphological methods [42]. To avoid the acid tolerance response in *Listeria monocytogenes* bacteria, we tried different approaches, one of which was based on applying the enzyme trypsin. Thus, trypsin was utilised to remove the *Listeria monocytogenes* bacterium template from the NIP-Ppy/SPCE and MIP-Ppy/SPCE. Accordingly, the electrodes were individually treated with solutions containing trypsin (Figure 5C) and L-lysine (Figure 5D). The current density for MIP-Ppy/SPCE, treated with trypsin, increased around three times compared with that registered for NIP-Ppy/SPCE, while MIP-Ppy/SPCE treated with L-lysine showed no changes in current density. Electrochemically registered results reveal that the electrical capacitance changed after removing imprinted bacteria; acetic-acid- and trypsin-based solutions were the most suitable for extracting entrapped *Listeria monocytogenes* bacteria and the preparation of MIP-Ppy. 

#### 3.3.2. Assessment of MIP-Ppy/Pt- and NIP-Ppy/Pt-Based Electrodes

**Figure 6:**
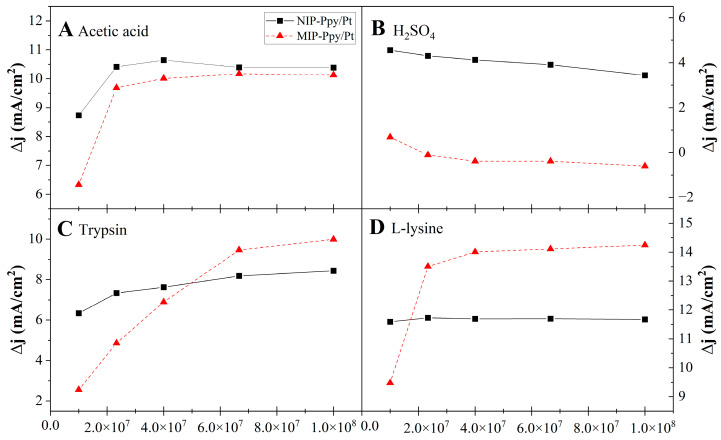
The current density of NIP-Ppy/Pt (solid black lines) and MIP-Ppy/Pt (dashed red lines) electrodes registered using pulsed amperometric detection after incubation in solutions containing different *Listeria monocytogenes* bacteria concentrations; *Listeria monocytogenes* from MIP-Ppy was extracted using different extraction solutions: (**A**) 10% acetic acid, (**B**) 0.05 M of sulphuric acid, (**C**) 10 U/mL of trypsin, (**D**) 0.1% L-lysine.

After preparation, NIP-Ppy/Pt and MIP-Ppy/Pt electrodes were incubated in solutions of *Listeria monocytogenes* bacteria of different concentrations and evaluated using pulsed amperometric detection based on 10 potential pulses of +600 mV for 2 s and 0 mV for 2 s. Figure 6A–D depict the dependence of the amperometric response of the second electrochemical system after the incubation of MIP-Ppy- and NIP-Ppy-modified platinum electrodes in PBS, pH 7.4, with a different concentration of *Listeria monocytogenes* bacteria.

With different working electrodes, including platinum, different techniques can be used to remove the imprinted material. Various solvents are often used to remove bacteria, for instance, acetic acid, hydrochloric acid, other acids, methanol, and mixtures of those solvents [32,34,43]. Although challenging, extraction of the bacteria from the polymer is essential for forming the MIPs [44]. During the formation of the MIP-Ppy structure within Ppy, entrapped *Listeria monocytogenes* bacteria were removed, leaving imprinted cavities using different extraction solutions, including 10% acetic acid (Figure 6A), 0.05 M of sulphuric acid (Figure 6B), 10 U/mL trypsin (Figure 6C), and 0.1% L-lysine (Figure 6D). Unlike the MIP-Ppy/SPCE electrode, a somewhat different situation was seen with the MIP-Ppy/Pt electrode. Trypsin- and L-lysine-based solutions proved to be the best for extracting *Listeria monocytogenes* bacteria from the Ppy layer. In the latter case, the current density of the MIP-Ppy/Pt electrode was twice as high compared to that registered by the NIP-Ppy/Pt electrode (Figure 6D). Trypsin proved to be effective in preparing the MIP-Ppy/Pt electrode suitable for the determination of *Listeria monocytogenes* bacteria in a broad concentration range.

### 3.4. Determination of Limit of Detection and Limit of Quantification

**Figure 7:**
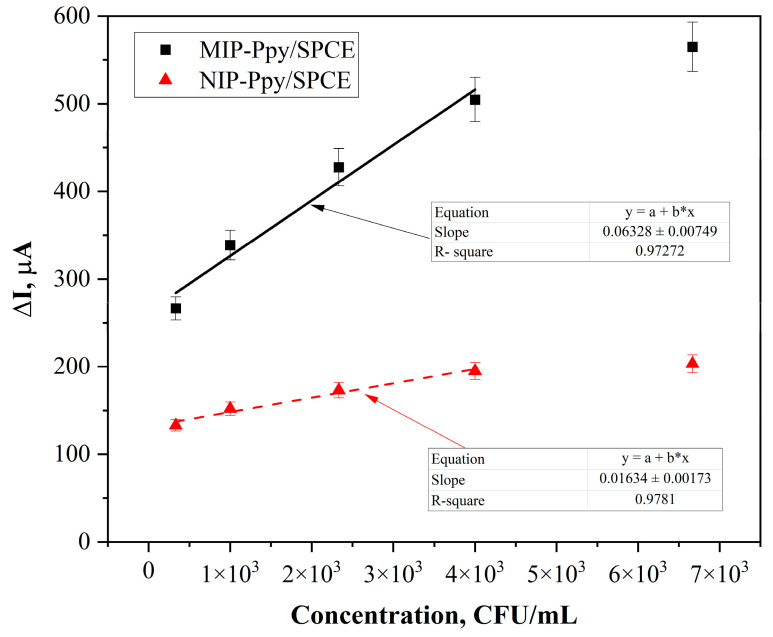
Calibration curve Δ*I* registered by MIP-Ppy/SPCE (black line) and NIP-Ppy/SPCE (red line) vs. *Listeria monocytogenes* concentration. Error bars are calculated as a percentage standard error.

As discussed, acetic acid and trypsin-based solutions were the most suitable for extracting *Listeria monocytogenes* bacteria entrapped within the Ppy-based layer and forming MIP-Ppy/SPCE. To assess the limit of detection (LOD) and limit of quantification (LOQ), pulsed amperometric detection-based electrochemical measurements were conducted. Δ*I* values were employed, respectively, for NIP-Ppy/SPCE and MIP-Ppy/SPCE, as analytical signals. *Listeria monocytogenes* bacteria concentration calibration logarithmic curves plotted against Δ*I* (µA) are shown in Figure 7. The slope for the variations in the current (I, µA) vs. concentration of *Listeria monocytogenes* bacteria (concentration expressed in CFU/mL) registered by the NIP-Ppy/SPCE electrode was 0.016 µA/(CFU/mL), with R^2^ = 0.98, while the linear regression slope for the *Listeria monocytogenes* bacteria imprinted MIP-Ppy/SPCE was 0.063 µA/(CFU/mL), with R^2^ = 0.97.

Molecular imprinting is ranked according to the relationship between the MIP and the non-imprinted polymer (NIP), which is obtained according to Equation (3) [45,46]:

(3) $$\mathrm{IF}=I_{\mathrm{MIP}}/I_{\mathrm{NIP}}$$

Usually, IF is called an imprinting factor, whereas Ayerdurai et al. [47] argued that an apparent imprinting factor is a more correct term for IF. According to the measurements, the MIP-Ppy/SPCE had an apparent imprinting factor toward the *Listeria monocytogenes* bacteria that was approximately four times higher than that registered by the NIP-Ppy/SPCE electrode. The LOD and the LOQ were calculated according to Equations (1) and (2). It was evaluated that the LOD and LOQ for the MIP-Ppy/SPCE were 70 CFU/mL and 210 CFU/mL, respectively, in the linear range from 300 to 6700 CFU/mL.

A comparison of electrochemical methods previously used to detect *Listeria monocytogenes* is shown in Table 1.

The investigation of the interaction between *Listeria monocytogenes* bacteria and the MIP-Ppy-modified electrode has several advantages, but the one that stands out the most is that just two of them use electrochemical techniques (Table 2). This suggests that there were only a few studies on applying MIP-based sensors for detecting *Listeria monocytogenes* bacteria. Additionally, Table 2 summarises other MIP-based sensors for certain bacterial species, employing electrochemical and quartz crystal microbalance (QCM) approaches. The electrode, the polymer used for MIP preparation, the bacteria extraction method, the analytical method, and sensitivity (LOD and linear range) are included in Table 2. In most cases, when an enzyme was used to extract the template from the polymer during MIP-based sensor design, that enzyme was lysozyme. Meanwhile, a similar enzyme, trypsin, was employed in this study.

## 4. Conclusions

This study involved the electrochemical modification of two types of electrodes, namely, SPCE and Pt, with different Ppy layers, i.e., not imprinted (NIP-Ppy) and bacteria *Listeria monocytogenes* imprinted (MIP-Ppy). The pulsed amperometric detection method was used to evaluate the performance of MIP-Ppy/SPCE and MIP-Ppy/Pt electrodes. MIP-Ppy/SPCE electrodes were found to be more effective in detecting *Listeria monocytogenes* in terms of the substantial changes in current density. Furthermore, the study analysed the efficiency of various template extraction solutions on the sensor’s sensitivity. The results showed that the acetic acid solution was highly effective in removing imprinted bacteria from the MIP-Ppy layer. MIP-Ppy/SPCE exhibited at least 12 times higher current density than NIP-Ppy/SPCE. The current density increased around 3 times for MIP-Ppy/SPCE designed by extraction of *Listeria monocytogenes* bacteria with trypsin compared to changes in the current density registered by similarly treated NIP-Ppy/SPCE. Based on these results, it can be assumed that an efficient MIP-Ppy-based sensor can be designed by extracting bacteria using acetic acid and the proteolytic enzyme trypsin. The results showed that the limit of detection (LOD) and limit of quantification (LOQ) of the MIP-Ppy/SPCE prepared using trypsin were 70 CFU/mL and 210 CFU/mL, respectively, within the linear range of 300 to 6700 CFU/mL.

## Figures and Tables

**Table 1:** Summary of the electrochemical methods previously used for the detection of *Listeria monocytogenes*.

Electrode	Detection Technique	Method Used	LOD, LOQ and LR	Ref.
Gold disk	RAA-based E-CRISPR ^1^	Square wave voltammetry	LOD 26 CFU/mL; LR 2.6 × 10^1^ to 2.6 × 10^9^ CFU/mL	[48]
Gold electrode	Sandwich assay	CV, EIS	LR 10^2^ to 10^6^ CFU/ml	[18]
Multiwalled carbon nanotube electrode	Immunoassay	CV	LOD 1.07 × 10^2^ CFU/mL; LR 10^2^ to 10^5^ CFU/mL	[49]
Aluminium disc	Immunoassay	EIS	LOD 1.3 log CFU/mL LR 1.3 to 4.3 log CFU/mL	[50]

^1^ RAA-based E-CRISPR—recombinase-assisted amplification-based CRISPR/Cas12a into an E-DNA biosensor platform.

**Table 2:** Summary of the MIP-based sensors for *Listeria monocytogenes* and other bacteria.

Bacteria	Electrode	Polymer	Bacteria Extraction from the Polymer	Method Used	LOD, LOQ and Linear Range (LR)	Ref.
*Listeria monocytogenes*	-	Acryloyl-functionalised chitosan with CdTe quantum dots	10% acetic acid, 1% SDS, water, and methanol	Fluorescence microscope	LOD 10^3^ CFU/mL	[32]
*Listeria monocytogenes*	GCE	Poly(TPA)	SDS/AA (*w*/*v*, 5%) solution	DPV	LOD 6 CFU/mL; LR 10 to 10^6^ CFU/mL	[33]
*Listeria monocytogenes*	GCE with MXenes nanoribbon (Ti_3_C_2_T_x_R)	Poly(Th) with	0.5 M HCl	DPV	LOD 2 CFU/mL; LR 10 to 10^8^ CFU/mL	[34]
*Klebsiella pneumoniae*	SPE	Acrylamide-based polymer with carbon or gold or rGO	10% acetic acid for 30 min	CV in PBS with a redox probe	LOD of 0.012 CFU/mL and LOQ of 1.61 CFU/mL	[51]
*Klebsiella pneumonia*	ITO coated glass electrode	Ppy obtained by the interfacial oxidative polymerisation process	DI and ethanol	DPV and CV in PBS with a redox probe	LOD of 1.352 CFU/mL	[52]
*Salmonella*	GCE	Ppy with MXene	SDS/acetic acid (5%, *w*/*v*) for 5 min and washed three times	EIS	LOD of 23 CFU/mL	[53]
*Pseudomonas aeruginosa*	QCM electrode	Overoxidised Ppy	With lysozyme (10 mg/mL) for 2 h at 4 °C and 10% Triton X for 80 min	QCM	LOD 10^3^ CFU/mL	[54]
*Escherichia coli* and *Pseudomonas aeruginosa*	QCM electrode	Overoxidised Ppy	Lysozyme (10 mg/mL) containing 10% Triton X and EDTA (200 µg/mL) for 1 day at room temperature	QCM	-	[55]
*Escherichia coli* (serotypes O157:H7 and O26:H11)	QCM sensor	Overoxidised Ppy	Lysozyme (30 mg/mL) and 5% SDS for 48 h at 30 °C	QCM	-	[56]
*Escherichia coli*	Gold electrode	Polymer of MAH, HEMA, and EGDMA	10 mM sodium phosphate buffer (pH 7.4) and treated with 10 mg/mL lysozyme solution (in 10 mM Tris-HCl buffer, pH 8.0, with 1 mM EDTA) for 30 min	Capacitance measurements in a continuous flow system	LOD 70 CFU/mL, LR 1.0 × 10^2^–1.0 × 10^7^ CFU/mL	[57]
*Escherichia coli* K-12	GCE	Polymer of 2-APBA and ANI	2 h treated with 2 mg mL^−1^ lysozyme enzyme in PBS (pH ¼ 7.4), 10% Triton X, water, and then overoxidised	DPV, EIS	-	[58]
*Saccharomyces cerevisiae* (Bakers’ yeast)	QCM	Polyurethane	Hot water	QCM	LOD 1 × 10^4^ cells/mL	[59]
*Staphylococcus epidermidis*	Gold electrode	Poly(3-APBA)	30 min with fructose (20 mM), plenty of water, and phosphate solution (pH 2.2) for 20 min	EIS	LR 10^3^–10^7^ CFU/mL	[60]
*Bacillus subtilis* endospore	GCE	polypyrrole/poly(3-methylthiophene)	In DMSO for 10 min at room temperature	CV and EIS	-	[61]

SPE—screen-printed electrode; rGO—reduced graphene oxide; redox probe—K_4_[Fe(CN)_6_]/K_3_[Fe(CN)_6_]; ITO—indium tin oxide; DPV—differential pulse voltammetry; GCE—glassy carbon electrode; SDS—sodium dodecyl sulphate; 3-APBA—3-aminophenylboronic acid; GCE—glassy carbon electrode; MAH—N-methacryloyl-L-histidine methylester; HEMA—2-Hydroxyethyl methacrylate; EGDMA—ethyleneglycol dimethacrylate; 2-APBA—2-aminophenylboronic acid; ANI—aniline; TPA—3-thiopheneacetic acid; SDS—sodium dodecyl sulphate; AA—acetic acid; Th—thionine; GCE—glassy carbon electrode.

## Data Availability

No additional information is available for this paper.
